# Edible Bird’s Nest Supplementation Improves Male Reproductive Parameters of Sprague Dawley Rat

**DOI:** 10.3389/fphar.2021.631402

**Published:** 2021-03-30

**Authors:** Farah Hanan Fathihah Jaffar, Khairul Osman, Chua Kien Hui, Aini Farzana Zulkefli, Siti Fatimah Ibrahim

**Affiliations:** ^1^ Department of Physiology, Faculty of Medicine, Universiti Kebangsaan Malaysia (UKM), Cheras, Kuala Lumpur, Malaysia; ^2^ Forensic Science Program, Faculty of Health Sciences, Universiti Kebangsaan Malaysia (UKM), Bangi, Selangor, Malaysia

**Keywords:** edible bird nest, sperm concentration, sperm motility, testosterone, follicle stimulating hormone (FSH), leutinizing hormone (LH)

## Abstract

Edible bird’s nest (EBN) is reported to have a positive *in vitro* proliferative effect and contain male reproductive hormones. Spermatogonia cells proliferate during spermatogenesis under male reproductive hormones stimulation that include testosterone, follicle-stimulating hormone (FSH), and luteinizing hormone (LH). Characterization of EBN through liquid chromatography-mass spectrometry (LCMS) has found testosterone as a base peak. Six types of amino acids, estradiol and sialic acid were among the major peaks that have been characterized. Based on the presence of these reproductive components, this study evaluated different doses of EBN on sperm parameters and male reproductive hormones of Sprague Dawley rats. Sixteen Sprague Dawley rats at the age of eight weeks were randomly and equally divided into four groups, which are Control, 10 mg/kg BW/d 50 mg/kg BW/d, and 250 mg/kg BW/d EBN group. The rats were fed with EBN enriched pellet daily and water ad-libitum. Rats were sacrificed and the organ was weighed for organ coefficients after eight weeks of treatment. Sperm concentration, percentage of sperm motility, and sperm viability were evaluated. Meanwhile, ELISA method was used to measure testosterone, FSH, and LH. Findings showed that there were no significant differences in organ coefficient between groups. Supplementation of 250 mg/kg BW/d EBN demonstrated a significant increase in sperm concentration, percentage of sperm motility as well as FSH and LH level compared to 10 mg/kg BW/d group. There was a dose-dependent increase in testosterone level but was not significant between groups. Based on these findings, EBN is concluded to have crucial effects on male reproductive parameters.

## Introduction

Edible bird’s nest (EBN) has been a delicacy in Chinese traditional medicine since the 16th century (Medway, 1969). The nest was partly built by the male swiftlet from the specialized salivary glands secretion of *Aerodramus fuciphagus* during the breeding season (Medway, 1962) and is found dominantly in South East Asia (Phach and Voisin, 2007; Aowphol et al., 2008; Babji et al., 2015; Manchi, 2015).

The major nutritional composition of EBN was reported to consist of protein followed by carbohydrate, fat, ash, and some moisture (Marcone, 2005; Hamzah et al., 2013; Saengkrajang et al., 2013). Besides, EBN contains high sodium and calcium content despite the presence of other mineral elements, which include magnesium, potassium, phosphorus, and iron. Essential amino acids like histidine, isoleucine, leucine, lysine, phenylalanine, methionine, threonine, and valine have also been detected and mentioned by various researches (Marcone, 2005; Saengkrajang et al., 2013). All these nutritional contents might contribute to a remarkable therapeutic potential of EBN.

The therapeutic potential of EBN has been scientifically proven including anti-osteoporosis effect (Matsukawa et al., 2011), osteoarthritis treatment (Chua et al., 2013), and the ability to enhance the immune system (Zhao et al., 2016). Moreover, EBN supplementation also has been proven to improve metabolic diseases (Yida et al., 2015), accelerate wound healing (Abidin et al., 2011), improves the length and weight of uterine and vaginal in ovariectomized rats (Zhiping et al., 2015) as well as improves the cognitive function of ovariectomized rats (Zhiping et al., 2015).

Despite the vast scientific exploration of EBN therapeutic potential, evaluation of its effect on male reproductive system is still lacking. Evaluation of EBN supplementation of the male reproductive system is important for two main reasons. First, due to its proven positive proliferative effect in human adipose-derived stem cells (hADSCs) and normal human fibroblasts (NHFs) (Roh et al., 2012). This positive effect may also promote the proliferation of spermatogonia during the spermatogenesis event in the testis.

Secondly, Ma and Liu (2012) had discovered the presence of male reproductive hormones, which include testosterone, follicle-stimulating hormone (FSH), and luteinizing hormone (LH) in EBN (Ma and Liu, 2012). Another study was done by Ma et al. (2012) also demonstrated that EBN supplementation in castrated male rats had a significant increase in serum testosterone and LH. This finding suggested that EBN can become a potential erectile dysfunction treatment (Ma et al., 2012). To date, this is the only study that evaluates the association of EBN with the male reproductive system.

Based on this characteristic, EBN is postulated to have a favorable effect on the male reproductive system. Since there is a limited number of EBN studies related to male reproduction, it is imperative to evaluate the effect of EBN supplementation on male reproductive parameters.

## Materials and Methods

### EBN Source and Extraction Method

The EBN was obtained from an identified swiftlet’s house at Bera, State of Pahang, Peninsular Malaysia. The identified swiftlet’s house is referring to the house that has been well maintained, good sanitation condition for high-quality EBN production, and swiftlet population is at minimum 2000 birds habitat in the house.

A single batch of EBN Extract was prepared from the collected EBN and used for the whole project. The whole extraction procedures are briefly described as follows:The EBN was cleaned from its impurities and dried at 50°C for 24 h before grounded and filtered with a 600 μm pore size mesh. The grounded EBN was then kept in distilled water (20 g/L water) at 25°C for 12 h before heated at 95°C for 120 min. The supernatant was filtered using Grade 1 Whatman filter paper and the filtrate was freeze-dried later. The freeze-dried EBN extract was stored at 4°C until use.

### Orbitrap Liquid Chromatography-Mass Spectrometry Analysis of EBN Extract

This analysis was done to characterize the compounds presence in the EBN extract. Approximately 2 mg of EBN extract was dissolved in 1 ml of HPLC grade methanol. The solution was sonicated for 15 min and filtered through a membrane filter with pore size 0.22 μm before analysis. About 10 μL of the sample was injected into the AccelaTM UHPLC System (Thermo Scientific, San Jose, United States) equipped with quaternary pump, a build degasser, a PDA detector and an auto-sampler. Separation of the EBN sample was carried out by using A Luna Kinetex RP C18 column (2.6 μm, 2.1 mm I.D. x 150 mm) at a flow rate of 200 μL/min over 40 min by using acetonitrile: 0.1% formic acid in water as eluent. The step gradient and isocratic solvent composition was depicted in Supplementary Table S1.

The sample was analyzed by LTQ mass spectrometer (Thermo Scientific, San Jose, United States) with m/z spectrum ranged from 50 to 1000 was recorded in negative ionization mode. The setting of electrospray ionization modes was as follows: source accelerating voltage, 4.0 kV; capillary temperature, 350°C; sheath gas flow, 40 arb and auxiliary gas, 20 arb.

A reference standard of 18 types of amino acid (AAS18, analytical standard) was obtained from Sigma-Aldrich and used in this analysis. This reference standard was separated and analyzed under the same setting explained above.

### Animals

A total of 16 (N = 16) Sprague Dawley male rats at the age of 8 weeks with an initial average weight of 250 ± 50 g were acquired from Universiti Kebangsaan Malaysia (UKM) Laboratory Animal Research Unit (LARU). They were divided randomly into four groups with four rats (n = 4) in each group. The first group was the Control group which was only fed with a standard food pellet. The other three groups received 10 mg/kg BW/d, 50 mg/kg BW/d and 250 mg/kg BW/d EBN extract respectively. The doses were supplied daily for eight consecutive weeks. The lowest EBN dose applied in this setting was based on the study done by Ma et al. (2012).

The rats were housed individually in ventilated cage (IVC), clean water was supplied *ad libitum* and kept at 12-h light: dark cycle in a room. Ambient temperature was maintained at 22 ± 5°C throughout the experiment. After eight weeks of treatment, rats were sacrificed by anesthetizing intraperitoneally with an overdose mixture of ketamine (3.34 mg⁄kg, Ilium, USA), xylazine (3.34 mg⁄kg, Ilium, United States) and Zoletil-50 (1.66 mg/kg, Virbac). The death of the animal was confirmed by the loss of righting reflex, cessation of heartbeat, loss of tail pinch reflex, loss of a pedal withdrawal reflex in the forelimbs and hindlimbs and lack of corneal reflex.

All animal procedures were approved by The National University of Malaysia (UKM) Animal Ethics Committee with the approval number FISIO/PP/2018/SITIFATIMAH/28-MAR./908-MAR.-2018-DEC.-2020.

### EBN Supplementation

EBN supplementation to the animals was done by natural feeding. This was done by enriching the food pellet with EBN extract. Enriching was done by mixing the dried EBN extract powder in a 100 g ground rats’ food pellet. The mixing weight was standardized at 100 g to ensure it is well-mixed. Following this, 100 ml of sterile tap water was added to mix it further. It was shaped into a pellet, and dried overnight at 60°C.

For every EBN doses, rats’ food pellet was enriched with 2 mg/g (10 mg/kg BW/d), 10 mg/g (50 mg/kg BW/d) and 50 mg/g (250 mg/kg BW/d) respectively. To supply daily doses to the animals, the following formula was applied:

$$\frac{\text{Daily dose}\left(\text{mg}/\text{kg}\right)\times \text{Weight of an animal}\left(\text{kg}\right)}{\text{Concentration of EBN enriched food pellet}\left(\text{mg}/\text{g}\right)}=\text{Daily amount of EBN enriched food pellet}\left(\text{g}\right).$$

The amount of EBN enriched food pellet that needs to be given daily to achieve daily doses was provided first for each animal to finish. The supply of normal food pellets was then given after all the EBN enriched food pellets were taken by the animals.

### Serum Sampling

Blood was drawn immediately from retro-orbital sinus following sacrifice. Blood was collected in BD Vacutainer SST II Advance Plus Blood Collection Tube (BD, United States) and left undisturbed for at least 30 min at room temperature. The collection tubes were then transferred into the centrifuge and serum was separated by centrifugation at 1500 *g* for 10 min at 4°C. Serum yields were aliquoted and stored at −80°C until analysis.

### Evaluation of Reproductive Organ Coefficient

Both sides of the testes and epididymis as well as seminal vesicle were carefully dissected and cleaned from surrounding adipose tissue before accurately weighed. The organ coefficient of each dissected reproductive organ was expressed according to the equation: the wet weight of organ (g)/body weight (g) x 100 (Feng et al., 2015).

### Epididymal Sperm Collection

Cauda epididymis was dissected out and minced in 2 ml of pre-warmed PBS (ThermoFisher Scientific, United States). Epididymal sperm were collected following 40-min incubation at 37°C to allow sperm to swim out of the epididymal tubules.

### Evaluation of Sperm Parameters

#### Sperm Concentration

A drop of 10 µl sperm suspension was placed on Makler Chamber (Sefi Medical Instruments Ltd. Haifa, Israel). Sperm concentration was counted as an average of five rows under 10x magnification of bright field microscope (Olympus CH-2, Japan).

#### Sperm Motility

Sperm motility was determined according to the World Health Organization (WHO) (WHO, 2010) recommendations. The sperm motility was categorized into three categories which are A: progressive motile sperm, B: non-progressive motile sperm and C: immotile sperm. A drop of 10 µl sperm suspension was placed on a microscope slide and cover slipped. A total of 200 cells were counted in duplicate under 40x magnification bright field microscope (Olympus CH-2, Japan). The sperm motility was reported as the percentage of total motile sperm (A + B/total counted sperm) x 100.

#### Sperm Viability

Evaluation of sperm viability was done by using the hypo-osmotic swelling test (HOST). The sperm suspension was mixed with a hypo-osmotic swelling solution in a ratio of 1:10. The hypo-osmotic swelling solution was prepared by adding 0.735 sodium citrate dehydrate (Sigma Aldrich, Germany) and 1.351 g d-fructose (Sigma Aldrich, Germany) in 100 mL distilled water. The mixture was incubated at 37°C for 30 min. About 10 µl from the mixture was placed on the microscope slide, smeared and let dry at room temperature.

In order to enhance the visibility of the sperm under bright field microscope visualization, the smear was stained with Diff Quick staining where the slides were dipped in Diff Quick Fix, Diff Quick 1 and Diff Quick II for 5 min respectively. Following this, the slides were rinsed and room dry. The viable sperm were then counted under 40 × magnification out of 200 sperm cells. Counting was done in duplicate.

### Determination of Testosterone, FSH and LH by ELISA

Serum testosterone was measured by competitive enzyme-linked immunosorbent assay (ELISA, Elabscience, Wuhan, China). On the other hand, serum FSH and LH were measured by sandwich ELISA (Elabscience, Wuhan, China). The intra-assay and inter-assay variability (CV) for all the ELISA kit was less than 10%.

All the assay procedures were done according to the kit instruction. Briefly, 50 µl standard and serum were pipetted to the 96 well testosterone ELISA plate in duplicate. Immediately, 50 µl biotinylated antibody was added into the well, sealed and incubated for 45 min at 37°C.

For FSH and LH hormone analysis, 100 µl standard and serum were pipetted to the 96 well of respective ELISA plate in duplicate. The plate was sealed and incubated for 90 min at 37°C and all the liquid was removed before immediately adding 100 µL respective biotinylated antibodies into the well. This was further followed up by incubation for 1 h at 37°C.

Following incubation with respective biotinylated antibody, all the solution was removed from the well before being washed three times. About 100 µl horseradish peroxidase (HRP) conjugated Avidin was added exclusively into each well. Further incubation was done for another 30 min at 37°C before being washed five times. Color development was done by adding 90 µl Substrate Reagent, covered from light and further incubated for another 15 min. The reaction was stopped by adding 50 µL Stop solution and the absorbance was measured at 450 nm.

Testosterone, FSH and LH serum levels were interpolated from 8 points standard curve plotted according to a 4-parameter logistic (4 PL) by using MyAssays.com.

### Statistical Analysis

Statistical analysis was performed using SPSS version 22.0 software (SPSS Inc., Chicago, IL, USA). One-way analysis of variance (ANOVA) followed by Dunnet and Tukey’s post-hoc analysis was conducted owing to the normal distribution and variance homogeneity of the data. A *p*-value < 0.05 was considered statistically significant.

## Results

### Characterization of the Compound in EBN Extract

Orbitrap LCMS analysis of EBN extract showed 20 major peaks (Supplementary Figure S1). From the chromatogram, it was found that peak number 3 showed a relative abundance of 100%. Characterization of this peak revealed that it is most likely to represent testosterone hormone. Other reproductive hormones that has been identified are estradiol and progesterone. Sialic acid is also among the compound that has been characterized. Besides, at least six types of amino acids were present as well as vitamin K and ionic compounds of calcium and magnesium iodide. Characterization of all probable compound from the LCMS data is depicted in Supplementary Table S2.

### Organ Coefficient

Findings showed that there was no statistically significant difference in organ coefficient between all the groups (Supplementary Table S3).

### Sperm Concentration, Motility and Viability

Sperm concentration showed a gradual increase from 10 mg/kg BW/d EBN to 250 mg/kg BW/d EBN group (Supplementary Figure S2A). However, there was no significant difference in sperm concentration between the Control, 10 mg/kg BW/d EBN and 50 mg/kg BW/d EBN group. On the other hand, the sperm concentration in the 250 mg/kg BW/d EBN group showed a significant increase compared to Control and 10 mg/kg BW/d EBN group with *p* < 0.05.

For the percentage of total motile sperm, there was also no significant difference between the Control, 10 mg/kg BW/d EBN and 50 mg/kg BW/d EBN group. However, the percentage of total motile sperm in the 250 mg/kg BW/d EBN group showed significant increased compared to the 10 mg/kg BW/d EBN group (*p* < 0.05) (Supplementary Figure S2B).

On the other hand, the 50 mg/kg BW/d EBN group showed significant difference compared to the Control group with *p* < 0.05 in the percentage of viable sperm. There was no significant difference in the percentage of viable sperm between the Control group, 10 mg/kg BW/d EBN and 250 mg/kg BW/d EBN group (Supplementary Figure S2C).

### Testosterone, FSH and LH Hormone Analysis

Testosterone, FSH and LH level in serum of Sprague Dawley rats showed a similar trend of gradual increase with increasing EBN dose. However, testosterone levels showed no significant difference between the groups (Supplementary Figure S3A). On the other hand, the 250 mg/kg BW/d EBN group showed a significant increase of FSH (Supplementary Figure S3B) and LH (Supplementary Figure S3C) compared to the 10 mg/kg BW/d EBN group where *p* < 0.05.

## Discussion

Traditionally, the consumption of EBN is claimed to increase libido. Libido in men usually is affected by a multitude of factors but it is closely regulated by the testosterone hormone (Allan et al., 2008; Corona et al., 2014; Rizk et al., 2017). Thus, this traditional claim of EBN on libido may be due to the presence of testosterone hormone in the EBN and its effect on promoting the testosterone release.

In this study, characterization of the major peak in Orbitrap LCMS analysis of EBN extract has identified the presence of several reproductive hormones. This includes testosterone, progesterone, and estradiol. However, the presence of FSH and LH were not detectable among the major peak. Testosterone, FSH, and LH are the well-known male reproductive hormones that play a vital role in controlling the complex process of spermatogenesis (Silber, 2018). Therefore, this study further evaluated the effect of EBN supplementation on these three hormones through serum hormonal analysis. It was found that there was a dose-dependent increase of testosterone, FSH, and LH which might suggest a direct correlation to the hormonal promoting effect of EBN.

Estradiol, another prominent reproductive hormone is also detected in our EBN extract. This is a predominant form of estrogen which is important for the female reproductive system. On the same note, estradiol also plays a critical role in a male sexual function where it is essential for modulating libido, erectile function, and spermatogenesis (Schulster et al., 2016). However, the testosterone and estradiol (T/E) ratio needs to be maintained above the cut-off point of 10 (Pavlovich et al., 2001). An increment of estradiol level which leads the T/E ratio to fall below the lower limit may cause decreased semen parameters (Raman and Schlegel, 2002).

Unfortunately, this study did not measure the serum estradiol level following EBN supplementation to clarify the T/E ratio. The evaluation of sperm parameters, however, reflects that EBN supplementation enhances spermatogenesis. This study found that there was a trend of gradual increase in sperm concentration and percentage of motile sperm with the increase of EBN dose. These positive findings in sperm concentration and motility reflected that major reproductive hormones were enhanced to the level that preserves and improves the spermatogenesis following EBN supplementation.

Despite the hormonal factors, the increment of sperm concentration might be related to the positive proliferative effect of EBN (Aswir and Wan Nazaimoon, 2011; Roh et al., 2012) that is highly associated with the presence of sialic acid (Aswir and Wan Nazaimoon, 2011). Since sialic acid is characterized as one of the major peaks, therefore, sialic acid might act synergistically with other factor presence in EBN to promote spermatogonia proliferation during spermatogenesis in a way yet to be understood.

EBN supplementation also supports the sperm maturation process by significantly increased the percentage of sperm motility and viability. Supplementation of 50 mg/kg BW/d EBN showed a significant increase in sperm viability compared to the Control group. On the other hand, 250 mg/kg BW/d EBN demonstrated a slight decrease in the percentage of viability without a statistically significant difference compared to 50 mg/kg BW/d EBN. A discrepancy in the result of sperm viability may probably due to the different membrane sensitivity of the epididymal sperm (Hall et al., 1991; Lagarrigue et al., 2020). The process of harvesting sperm from the cauda epididymis might be mixed up with the adjacent caput epididymis. Subsequently, this led to a mixture of different sperm maturity with immature sperm membranes and therefore demonstrated a different sensitivity toward the viability test (HOST). There is a high possibility that the percentage of sperm viability may show a different result if ejaculated sperm is used for evaluation.

Reduction in organ weight is the most prominent sign of testicular toxicity (Fallahzadeh et al., 2017; Nowrouzi et al., 2019). All EBN doses applied in this study, however, demonstrated no obvious testicular toxicity as the organ coefficient of the testes, epididymis, and seminal vesicle showed no significant difference between the groups. Therefore, overall study findings prove that EBN has a positive effect on the male reproductive parameters.

However, future study is needed to evaluate the protective and/or curative effect of EBN with various factors affecting male fertility. Furthermore, male infertility is often a multi-dimensional problem (Muhammad et al., 2015). Thus, the suitability of EBN supplementation in various factors affecting male fertility needs to be further evaluated. At this moment, EBN holds multiple factors which include reproductive hormones and the proliferative effect that help to improve male reproductive parameters. It is well known that EBN also has antioxidant properties (Yida et al., 2014; Zulkifli et al., 2019) that might benefit from oxidative stress associated with male infertility in the future. Therefore, EBN could be a good candidate to fit with the multi-dimensional problem of male infertility.

## Data Availability

The original contributions presented in the study are included in the article/Supplementary Material, further inquiries can be directed to the corresponding author.
