# Supplementary material for: Edible Bird’s Nest Supplementation Improves Male Reproductive Parameters of Sprague Dawley Rat
Source: Front Pharmacol. 2021 Mar 30;12:631402. doi: 10.3389/fphar.2021.631402 (PMC8111441; doi:10.3389/fphar.2021.631402)
Supplement: Supplementary file 1 [file DataSheet1.pdf]

Supplementary Material

Supplementary Tables

Supplementary Table 1 The step gradient and isocratic solvent composition for separation of EBN sample

Time (min)	Acetonitrile	0.1% Formic acid in Water
0	5.0	95.0
5	5.0	95.0
20	95.0	5.0
25	95.0	5.0
25	5.0	95.0
30	5.0	95.0

Supplementary Table 2 Identification of the major EBN chromatogram peak from LC-MS analysis

Peak	Rt (min)	m/z	Molecular weight (g/mol)	Molecular formula	Probable compound
1	1.51	118.000	117.15	$C_5H_{11}NO_2$	L-Valine
2	1.76	160.842	161.27	$C_6H_{15}NO_2Si$	Trimethylsilyl l-Alanine
3	11.53	371.218	372.5	$C_{23}H_{32}O_4$	Testosterone, 3,17beta-bisAc
4	12.58	385.233	386.5	$C_{24}H_{34}O_4$	Progesterone 16,17-acetonide
5	12.84	187.097	186.18	$C_8H_9NO_4$	4-Pyridoxic Acid-d3
6	13.73	201.113	202.25	$C_9H_{18}N_2O_3$	L-Leucyl-L-alanine
7	14.42	173.118	174.2	$C_6H_{14}N_4O_2$	L-Arginine
8	14.64	215.128	215.13	$C_6H_8F_3NO_4$	(2S)-3-Hydroxy-2-methyl-2-[(2,2,2-trifluoroacetyl)amino]propanoic acid
9	15.12	225.149	225.16	$C_8H_{10}F_3NO_3$	Proline, N-(trifluoroacetyl)-, methyl ester
10	15.48	229.144	231.29	$C_{11}H_{21}NO_4$	N-(Tert-Butoxycarbonyl)-L-leucine
11	15.48	229.144	231.29	$C_{11}H_{21}NO_4$	N-(Tert-Butoxycarbonyl)-L-leucine
12	16.31	243.16	243.21	$C_{10}H_{13}NO_6$	L-(+)-Threose, aldononitrile, triacetate
13	16.68	311.185	311.3	$C_{18}H_{17}NO_4$	Fmoc-beta-alanine
14	16.86	207.139	209.67	$C_{11}H_{12}ClNO$	Vitamin K5 hydrochloride
15	17.15	257.175	259.3	$C_{11}H_{21}N_3O_4$	L-Glutaminyl-L-isoleucine
16	17.32	207.139	207.23	$C_{11}H_{13}NO_3$	Glycine, N-(m-toluoy)-, methyl esterl
17	17.47	309.17	309.27	$C_{11}H_{19}NO_9$	Sialic acid
18	18	271.191	272.4	$C1_8H_{24}O_2$	Estradiol
19	18.12	293.175	293.89	CaI_2	Calcium iodide
20	18.96	277.18	278.114	I_2Mg	Magnesium iodide

Supplementary Table 3 Organ coefficient (wet weight of organ (g)/body weight (g)) x 100

Group/Organ Coefficient	Testes	Epididymis	Seminal Vesicle
Control	0.39 ± 0.02	0.16 ± 0.01	0.29 ± 0.01
10 mg/kg BW/d EBN	0.27 ± 0.04	0.13 ± 0.01	0.30 ± 0.05
50 mg/kg BW/d EBN	0.31 ± 0.02	0.13 ± 0.01	0.35 ± 0.03
250 mg/kg BW/d EBN	0.32 ± 0.03	0.14 ± 0.01	0.32 ± 0.04

Data are presented as Mean ± SEM with *n*=4. No significant different was noted between the groups for all organ coefficient.

Supplementary Figure

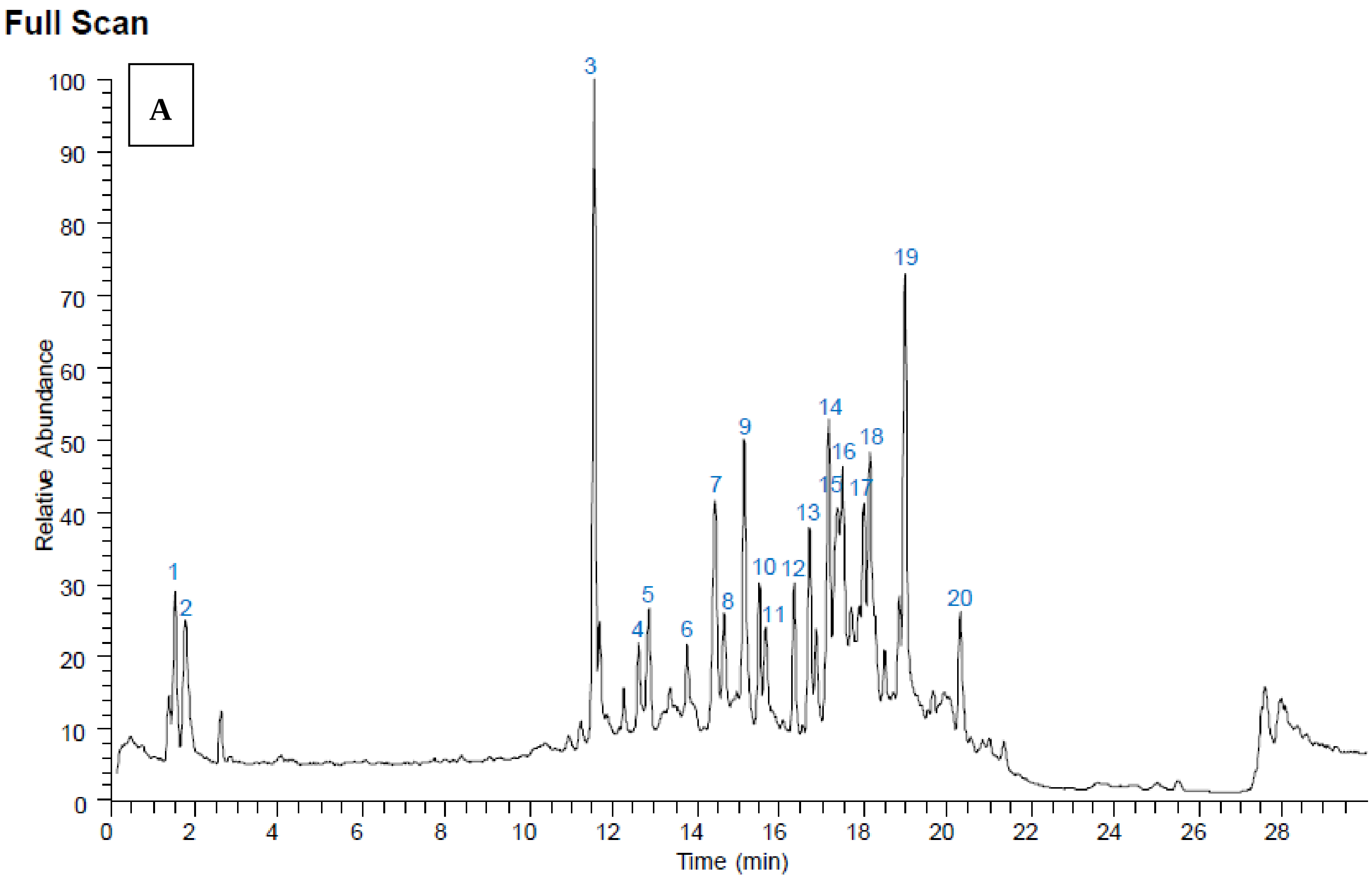

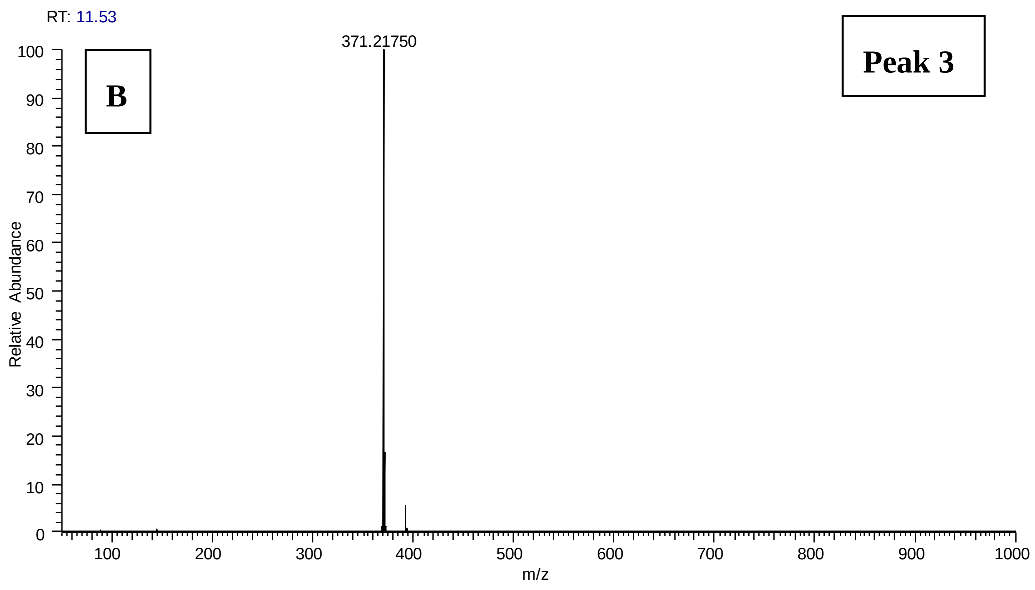

Supplementary Figure 1. Chromatogram of EBN compound characterization through LC-MS
A represents full scan chromatogram of Orbitrap LCMS analysis
B represents the most intensive peak in the mass spectrum

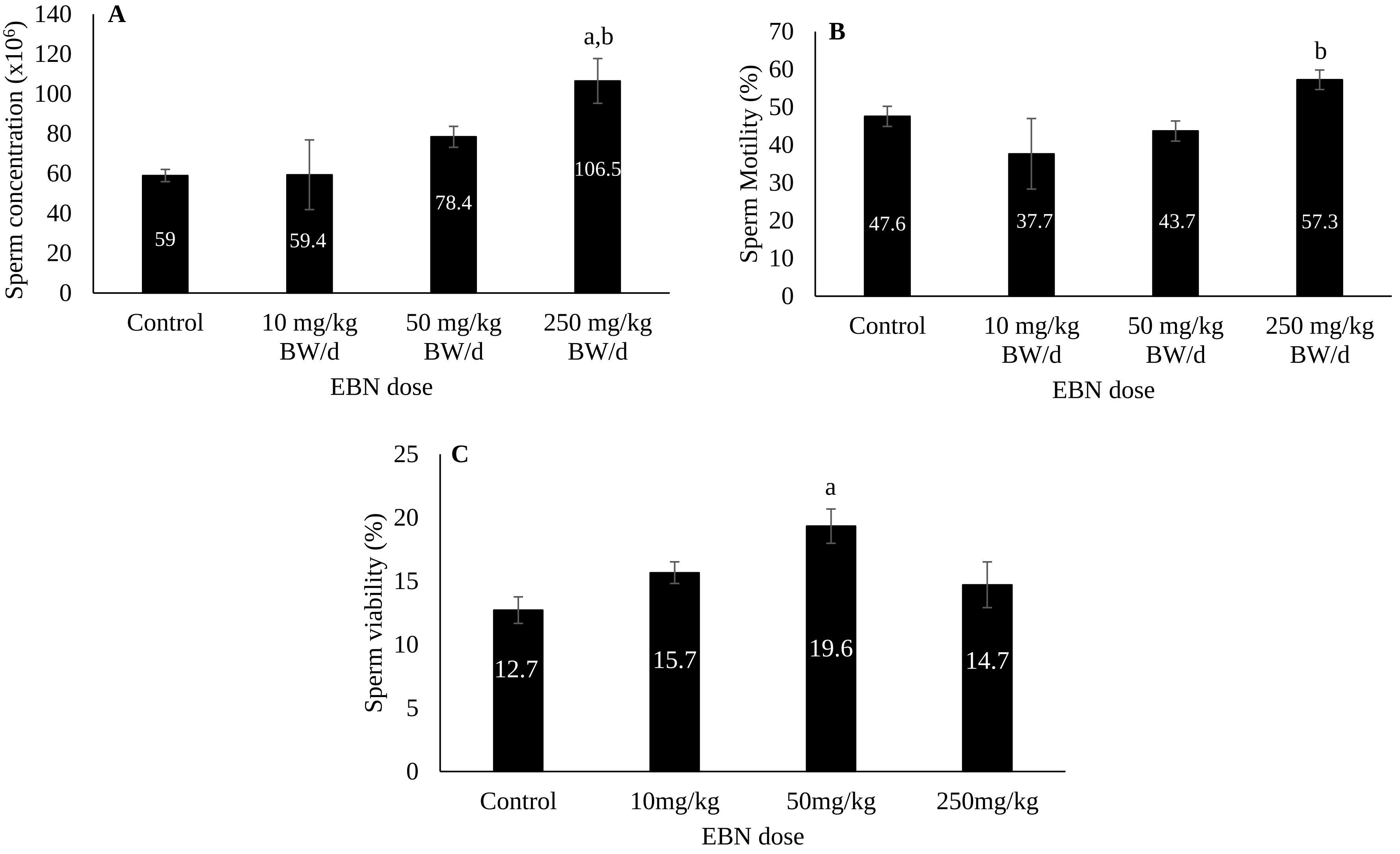

Supplementary Figure 2. The effect of EBN supplementation on sperm parameters.

A Sperm concentration with the respective dose of EBN treatment. Data are presented as Mean ± SEM with n=4. [a] represents a significant difference compared to the Control group with $p<0.05$; [b] represents a significant difference compared to the 10 mg/kg BW/d EBN group with $p<0.05$.

B Percentage of total motile sperm with the respective dose of EBN treatment. Data are presented as Mean ± SEM with n=4. [b] represents a significant difference compared to the 10 mg/kg BW/d EBN group with $p<0.05$.

C Percentage of viable sperm with the respective dose of EBN treatment. Data are presented as Mean ± SEM with n=4. [a] represents a significant difference compared to the Control group with $p<0.05$.

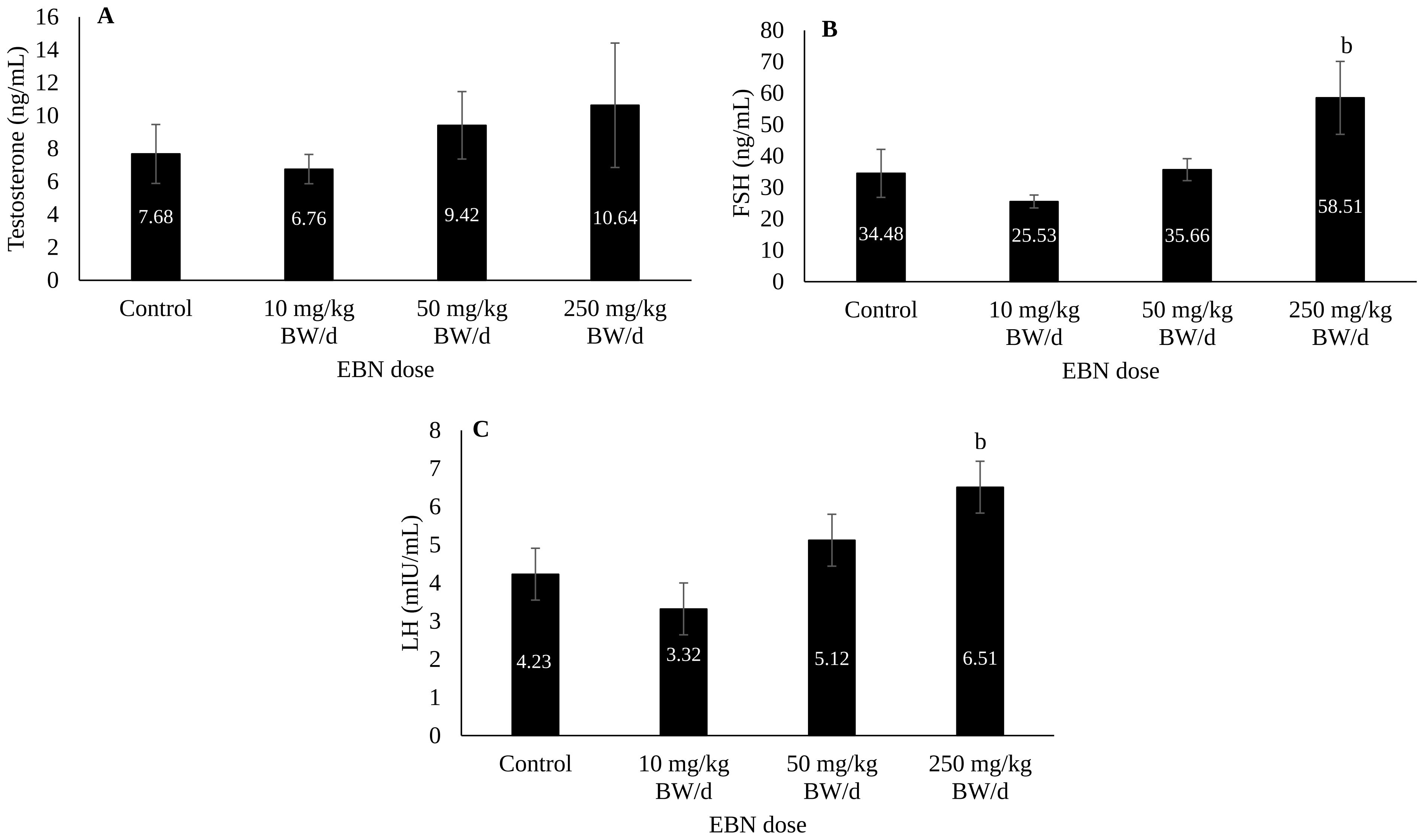

Supplementary Figure 3. The effect of EBN supplementation on serum hormonal level.

A Testosterone level in serum with a respective dose of EBN treatment. Data are presented as Mean ± SEM with n=4. No significant difference between the groups was noted.

B FSH level in serum with a respective dose of EBN treatment. Data are presented as Mean ± SEM with n=4. [b] represents a significant difference compared to the 10 mg/kg BW/d EBN group with $p<0.05$.

C LH level in serum with a respective dose of EBN treatment. Data are presented as Mean ± SEM with n=4. [b] represents a significant difference compared to the 10 mg/kg BW/d EBN group with $p<0.05$.
